# Warfarin-Associated Diaphragmatic Hernia: An Unusual Diagnosis

**DOI:** 10.1155/2015/987940

**Published:** 2015-05-20

**Authors:** Cristina Vilhena, Cátia Gameiro, Cláudia Tomás, Antónia Santos, Raquel Ilgenfritz

**Affiliations:** ^1^Department of Obstetrics and Gynecology, Garcia de Orta Hospital, Avenida Torrado da Silva, 2801-951 Almada, Portugal; ^2^Department of Anatomical Pathology, Garcia de Orta Hospital, Avenida Torrado da Silva, 2801-951 Almada, Portugal

## Abstract

Fetal warfarin syndrome is a consequence of maternal intake of warfarin during pregnancy and comprises a wide range of manifestations, including some typical facial dysmorphologic features. The authors report a case of prenatal ultrasonographic diagnosis of warfarin embryopathy in an obese woman on unsupervised warfarin prophylaxis at the 16th week of gestation. The fetus presented with facial dysmorphism, pectus excavatum, diaphragmatic hernia, and pulmonary hypoplasia. To the best of our knowledge, this is the second reported case of warfarin-associated diaphragmatic hernia.

## 1. Introduction

Di Sala syndrome or warfarin embryopathy is a condition resulting from fetal exposure to warfarin ingested by the mother during pregnancy [1, 2]. This is a rare condition with a widely reported incidence, ranging from 0% to almost 30% of exposed pregnancies, with an average risk of around 6% [1]. Several cases have been reported [3–5] and there is a wide range of manifestations including neonatal death and a dysmorphology in the neonate with characteristic classical features such as nasal hypoplasia and stippling epiphyses [6, 7]. Warfarin is a vitamin K antagonist used in the management and prevention of thromboembolic disorders that readily crosses the placenta due to its low molecular weight [8]. Fetal exposure to this drug during the first trimester appears to affect the synthesis of proteins crucial to bone and cartilage formation. In order to minimize the risk of warfarin embryopathy during this critical period, a commonly suggested regimen involves substituting warfarin with heparin. However, as pointed out by some authors, this strategy might be insufficient since the effect of warfarin can last for five days after the intake of the last tablet [9]. Prenatal diagnosis of warfarin embryopathy is difficult, and even high-detail ultrasonography may not detect the anomalies caused by warfarin use. Gupta et al. suggested that 3D ultrasound might add important information for detecting midfacial anomalies such as the depressed nasal bridge and midfacial hypoplasia of warfarin embryopathy [10]. However, as shown in a retrospective study by Wainwright and Beighton [6], there is a wide range of less well-known manifestations that might be related to warfarin exposure.

## 2. Case Report

**Figure 1:**
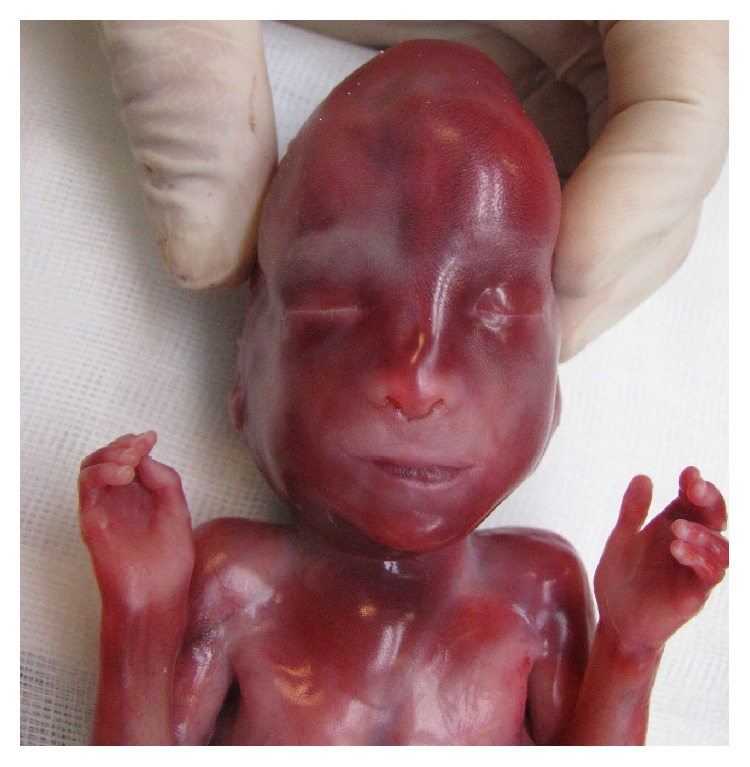
Anatomical postmortem exam of the fetus showing short nose with flat nasal bridge.

**Figure 2:**
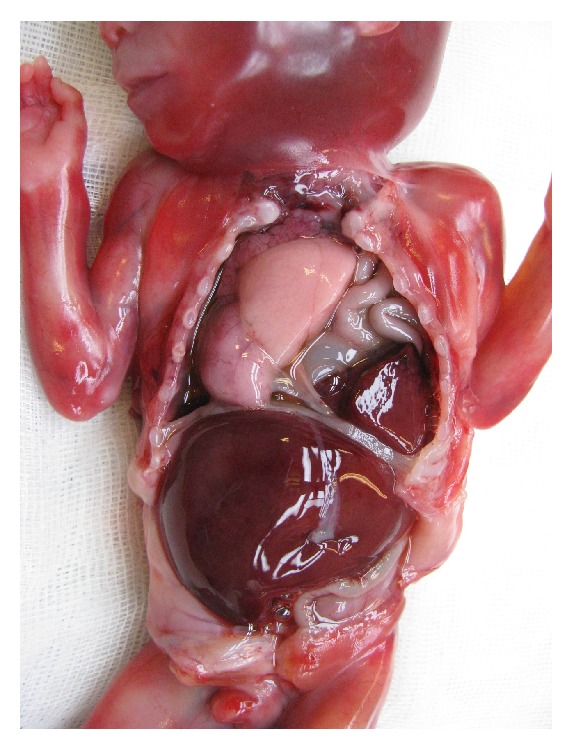
Anatomical postmortem exam of the fetus showing left diaphragmatic hernia with intrathoracic stomach, spleen, bowel, and liver.

**Figure 3:**
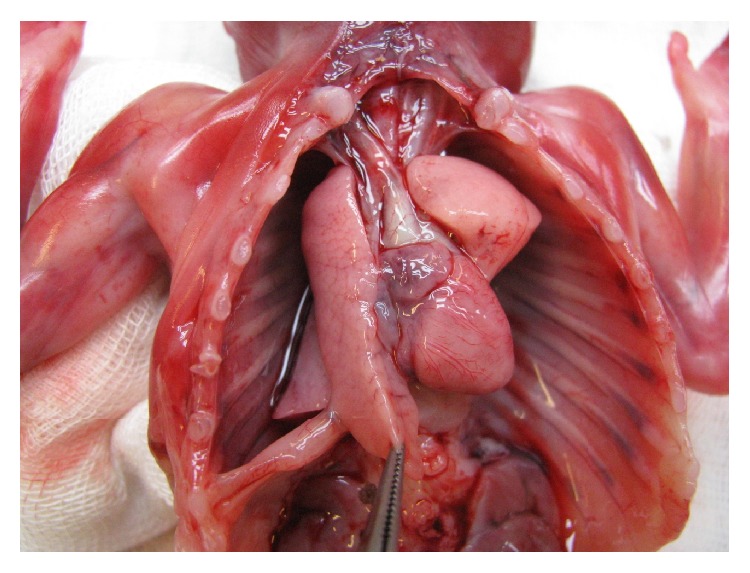
Anatomical postmortem exam of the fetus showing pulmonary hypoplasia and right heart axis deviation.

A 36-year-old woman with two previous term deliveries by cesarean section attended the prenatal clinic, at 16 weeks of pregnancy. She had morbid obesity (weight 165 kg; height 160 cm; BMI = 57.9 kg/m^2^) and recurrent superficial venous thrombosis initiated in 2011 following a traumatic injury in the left leg. At that time she was under oral combined contraception, which she kept until 2013 when she realized she was pregnant. Taking into account her multiple recurrent episodes of superficial venous thrombosis as well as her other risk factors such as hormonal contraception and obesity, she was prescribed warfarin by her vascular surgeon and she was monitoring the INR on an ambulatory day care center. Her previous screening for thrombophilia yielded negative results. Considering the known teratogenic effects of warfarin, the patient was advised, in the first prenatal visit, to switch to a therapeutic dosage of low molecular weight heparin (enoxaparin 100 mg id). A two-dimensional (2D) screening scan was performed at 18 weeks revealing the following features: nasal hypoplasia and left diaphragmatic hernia. Appropriate prenatal counseling was given regarding the possible causes of these ultrasonographic findings as well as advising the prognosis related to diaphragmatic hernia. Amniocentesis was performed and fetal karyotype was studied. Before fetal karyotype results were available, the couple requested pregnancy termination. To this end, the departmental protocol was followed administering a low dose of vaginal misoprostol tablets every six hours. A 217 g male fetus was delivered. The pathology exam revealed classical facial features of warfarin embryopathy: short nose with flat nasal bridge and perialar flatness. Pectus excavatum, pulmonary hypoplasia, right mediastinal shift, and left diaphragmatic hernia with intrathoracic stomach, spleen, bowel, and liver were also evident at fetal autopsy (Figures 1, 2, and 3). The placenta weighed 74 grams and showed no abnormalities. The fetal karyotype was normal (46, XY).

## 3. Discussion

The report describes a case of Di Sala syndrome or warfarin embryopathy presenting itself as typical facial dysmorphism, pectus excavatum, and diaphragmatic hernia. To the best of the authors' knowledge, this is the second case report of warfarin-induced diaphragmatic hernia [11]. The fact that this pregnant woman was under oral anticoagulation on the first trimester prompted a meticulous scanning seeking dysmorphologic features of warfarin embryopathy, which allowed early diagnosis. Ultrasonographic assessment was particularly difficult in this patient due to her visceral obesity. A series by Wong et al. found that, despite the high risk of fetal wastage, there was a relative low risk of major complications, except for some minor cosmetic defects such as nasal hypoplasia [2]. This is important to be taken into account at the moment of counseling the couple in the cases where facial dysmorphology is the only anomaly present. Unfortunately, that was not the case of this patient and the diagnosis of left diaphragmatic hernia with intrathoracic liver assigned a particular bad fetal prognosis. In light of the presently known mechanisms associated with warfarin embryopathy, the importance of an early switch to heparin has to be emphasized. Notwithstanding, the optimal anticoagulant strategy remains to be established. One of the most commonly suggested regimens involves substituting warfarin with heparin between the 6th and 12th gestation weeks [12]. In spite of this early switch, it is well known that warfarin has a long half-life, and substitution at the 6th week may be too late to avoid embryopathy, as recently suggested by Walfisch and Koren [9]. On the contrary, warfarin could be safely used after 12 weeks of gestation but should be discontinued after 34–36 weeks of gestation, and cesarean section should be considered in order to avoid birth trauma [8]. Even though it is infrequent to deal with pregnant women with conditions that wage a high risk of thrombosis (such as in the present case), one should be aware that fetal exposure to warfarin is teratogenic especially in the first trimester and the maternal risks of stopping or switching this medication must be balanced with the fetal risks involved.
